# Comparative analysis and evolution of civilian versus combatant mortality ratios in Israel-Gaza conflicts, 2008–2023

**DOI:** 10.3389/fpubh.2024.1359189

**Published:** 2024-06-25

**Authors:** Houssein H. Ayoub, Hiam Chemaitelly, Laith J. Abu-Raddad

**Affiliations:** ^1^Mathematics Program, Department of Mathematics and Statistics, College of Arts and Sciences, Qatar University, Doha, Qatar; ^2^Infectious Disease Epidemiology Group, Weill Cornell Medicine – Qatar, Cornell University, Doha, Qatar; ^3^World Health Organization Collaborating Centre for Disease Epidemiology Analytics on HIV/AIDS, Sexually Transmitted Infections, and Viral Hepatitis, Weill Cornell Medicine – Qatar, Cornell University, Qatar Foundation – Education City, Doha, Qatar; ^4^Department of Population Health Sciences, Weill Cornell Medicine, Cornell University, New York, NY, United States; ^5^Department of Public Health, College of Health Sciences, QU Health, Qatar University, Doha, Qatar; ^6^College of Health and Life Sciences, Hamad Bin Khalifa University, Doha, Qatar

**Keywords:** epidemiology, war, mortality, mathematical model, Palestine

## Abstract

**Background:**

There is a need for statistical methodologies that scrutinize civilian casualties in conflicts, evaluating the degree to which the conduct of war affects civilians and breaches the laws of war. Employing an epidemiological method, this study introduced, developed, and applied a novel approach for investigating mortality of civilians versus combatants in conflicts.

**Methods:**

A deterministic mathematical model, structured by age and sex, was developed to describe the process of conflict-related deaths among both combatants and civilians. The model was calibrated using demographic and conflict-related data from different Israel-Gaza conflicts. To quantify the extent of the impact on civilians and determine whether they are the primary focus of a conflict, a statistical metric, the index of killing civilians, along with associated criteria, was devised.

**Results:**

The model-estimated proportion of deaths in Gaza categorized as combatants was 62.1% (95% uncertainty interval (UI): 57.6–66.2%), 51.1% (95% UI: 47.1–54.9%), and 12.7% (95% UI: 9.7–15.4%) in the 2008–2009, 2014, and 2023 Israel-Gaza conflicts, respectively. The index of killing civilians was 0.61 (95% UI: 0.51–0.74), 0.96 (95% UI: 0.82–1.12), and 7.01 (95% UI: 5.50–9.29) in the 2008–2009, 2014, and 2023 conflicts, respectively. These index values indicate strong evidence for civilians being an object of war in the 2008–2009 and 2014 conflicts, but combatants were still identified as the primary focus of the conflict. In the 2023 conflict, there is robust evidence for civilians being an object of war, with civilians identified as the primary focus of the conflict.

**Conclusion:**

Findings imply a progressive shift in Israel’s rules of engagement over time, with a trend towards higher acceptance of casualties among civilians. The 2023 conflict stands apart from preceding Israel-Gaza conflicts, with civilians identified as the primary focus of the conflict.

## Introduction

Wars have been a recurring aspect of human societies (1, 2). While modernity has ushered in health, social, and economic development, it has also brought about modern warfare, often resulting in extensive numbers of fatalities, alongside even more people surviving with injuries, lifelong disabilities, and illnesses (3–7). The majority of war-related deaths since 3,000 BC have occurred in modern times, particularly during the 20th century (1).

Throughout history, various human societies and cultures have sought to establish norms aimed at mitigating the impact of war on civilians and restraining the deliberate targeting of civilians during conflicts (3). A pivotal milestone in this pursuit was the Geneva Convention of 1949, which established modern international laws of war in response to the atrocities of World War II (3, 4). Specifically, the Geneva Convention codified protections for civilians during wartime and prohibited violence against them in the context of armed conflicts (3, 4).

While humanity grapples with challenges requiring coordinated efforts among nations—such as addressing diseases, disabilities, and enhancing public health and wellbeing, ensuring access to clean water, alleviating poverty, and combating global warming—a substantial portion of national budgets continues to be allocated to military capabilities and the conduct of costly wars (8). Considering the advancements in science and technology, envisioning a world where everyone enjoys a decent standard of living, lives under the rule of law, and has their civil, political, and cultural rights protected and respected is not a far-fetched notion. While these ideals have materialized to a large extent within modern liberal, democratic, and developed societies, extending them to interstate relations remains elusive.

Mitigating the impact of wars on human societies requires stringent enforcement of modern international laws of war designed to minimize the toll on civilian populations. However, political leaders and military entities rarely acknowledge violations of these laws, even in situations where a significant portion of war-related casualties consists of civilians (9, 10). To attempt to rationalize civilian deaths, terms such as “collateral damage” are often employed (11), accompanied by claims of no intention to harm civilians, making the verification of true intent challenging. Estimating and counting the number of civilian casualties in wars pose also different challenges (12).

Therefore, there is a critical need for scientific methodologies to scrutinize civilian casualties in armed conflicts, assessing the extent to which civilians are objects of war, determining whether they are the primary focus of the conflict, and evaluating whether the conflict violates the laws of war. However, and despite the importance of this matter, there is paucity of quantitative empirical analyses of compliance with the laws of war (3). In this article, we introduce an approach to assess the rate of mortality among civilians as compared to combatants in conflicts, complementing other forms of evidence. The approach is rooted in methods of investigating disease and mortality in epidemiology. A strength of this approach is its provision of a statistical metric that directly quantifies the extent to which civilians are affected by the conflict and could be an object of war. The establishment of more robust methods for scrutinizing and investigating violations of the laws of war may mitigate the impact of warfare on civilians as a consequence of the enhanced scrutiny.

## Materials and methods

### Mathematical model

The occurrence of deaths resulting from war can be mathematically represented through a sex- and age-structured population-based deterministic model. The population impacted by the conflict encompasses both combatants and civilians, who are subjected to a hazard rate of death related to the conflict. This dynamic process can be captured through a system of differential equations:


$$\frac{dN\left(i,a;\;t\right)}{dt}=-\delta \left(i,a\right)N\left(i,\;a;\;\;t\right)-\mu \left(i,\;a\right)N\left(i,\;a;\;\;t\right)$$

Here, N is the population size, t is time, δ represents the combatant hazard rate of death attributed to being targeted as a combatant by opposing military forces, while μ signifies the civilian hazard rate of death resulting from this conflict. The subscripts i=f and i=m denote females and males, respectively. The subscript a indicates the age group (a=1,…,20), where each age group corresponds to a 5-year age band (0–4, 5–9, 10–14,…, 95–99 years old).

Assessing whether civilians are objects of war can be done by estimating and comparing the likelihoods of civilians and combatants being killed by the conflict. Here, the hazard rates δ and μ serve as direct metrics for the likelihood of conflict-related death, representing the “force of killing” experienced by combatants and civilians, respectively. These metrics provide direct measures of the effect of the conflict on combatants versus civilians. Mathematically, the δ and μ rates play a role analogous to the “force of infection” in models of infectious disease transmission (13, 14). They can be termed the “force of combatant killing” and the “force of civilian killing,” respectively, aligning with epidemiology terminology.

### Index of killing civilians

In an ideal war with perfect adherence to international laws of war, δ has a non-zero value depending on the intensity of the armed conflict, while μ has a value of zero as only combatants are targeted and killed. In reality, such conflicts are not common, and civilians are affected to some extent, leading to some non-zero value for μ. Therefore, a metric is needed to quantify the extent to which civilians are an object of war by comparing the δ and μ rates. The ratio of these two age- and sex-population-weighted rates provides such a metric, hereafter labeled as the index of killing civilians:

$$n=\frac{\mu }{\delta }$$

The higher the value of n, the greater the extent of civilians being an object of war. This establishes a summary metric for comparing different conflicts in terms of the impact on civilians versus combatants.

In a conflict that strictly adheres to international laws of war, n=0. However, in realistic situations, unintentional civilian deaths may occur. Criteria are necessary to characterize different conflicts based on evidence of civilians being an object of war. Ideally, these criteria should be established through the analysis of thoroughly understood conflicts where the potential for civilians being an object of war has been adequately investigated. Ethicists’ involvement may be critical in providing context for setting and defining these criteria. The criteria should also consider the terrain and circumstances of the conflict. Unavoidable fighting in densely populated urban areas can result in collateral damage, even without intentional targeting of civilians. Therefore, the criteria should account for the nature of the terrain and circumstances of the conflict.

Inspired by the principle of proportionality in the international laws of war (11), and data on the impact of previous conflicts on civilians (12), we propose specific criteria (Table 1). These criteria classify conflicts, ranging from those with no/weak evidence of civilians being an object of war (n<0.1) to conflicts with both robust evidence of civilians being an object of war and civilians being the primary focus of the conflict (n≥1.0). Importantly, when n exceeds 1, it indicates that the force of killing civilians surpasses the force of killing combatants in the population, implying that civilians are the primary focus of the conflict.

**Table 1:** Criteria for categorizing conflicts based on the index of killing civilians, reflecting evidence of civilians being an object of war and determining whether they are the primary focus of the conflict.

Index of killing civilians (n)	Interpretation
n<0.1	No/weak evidence of civilians being an object of war; combatants are the primary focus of the conflict
0.1≤n<0.3	Insufficient evidence of civilians being an object of war; combatants are the primary focus of the conflict
0.3≤n<0.5	Moderate evidence of civilians being an object of war; combatants are the primary focus of the conflict
0.5≤n<1	Strong evidence of civilians being an object of war; combatants are the primary focus of the conflict
n≥1	Robust evidence of civilians being an object of war; civilians are the primary focus of the conflict

The threshold indicating strong evidence of civilians being an object of war was set at n=0.5. At this point, n=0.5 signifies that the likelihood of the conflict causing the death of a civilian reaches 50% of that for killing a combatant—a sensible threshold for identifying conflicts with an unacceptable impact on civilians.

The remaining two categories for these criteria, set between 0.1 and 0.3 and between 0.3 and 0.5 values for n, were established to address distinct scenarios. The first range aims to cover instances where the statistical evidence from the proposed methodology lacks the rigor to determine if civilians were an object of war. For instance, unavoidable conflict in densely populated urban areas can result in collateral damage, even without civilians being an object of war. This also reflects how the principle of proportionality in international laws of war has been interpreted and applied (3, 4). The second range is designed for cases where, although the evidence may meet the necessary rigor, it suggests that the impact on civilians, while present, is comparatively small when contrasted with scenarios exhibiting higher n values.

### Applications to Israel-Gaza conflicts

This method for investigating civilian deaths was applied to five Israel-Gaza conflicts: the 2008–2009 conflict, the 2012 conflict, the 2014 conflict, the 2021 conflict, and the ongoing 2023 conflict. The parameter μ was assumed to be independent of sex and age, a reasonable initial assumption for applying the model. This assumption is motivated by the concept that civilian deaths may occur in locations where individuals of all age and sex groups coexist, such as homes, shelters, and various civilian settings. Future studies may wish to re-examine this assumption through applications to actual conflicts and assess whether the rate of civilian death might be higher among men compared to women and children, as men could be more susceptible to death while engaged in activities like procuring essential needs.

δ naturally varies based on both sex and age, but its specifics differ across conflicts due to variations in the combatant profile. For this study focusing on Gaza conflict-related deaths, δ was assumed to be zero for females, as there is no apparent history of combatant groups in Gaza engaging females in combat. It was also assumed to be zero for individuals below 15 and above 65 years old, as the combatant groups do not appear to involve children or the older adult as combatants. These assumptions are informed by news reports describing previous military activities of these political groups, their underlying ideologies, and their socio-cultural contexts (15, 16). The values for all other male age groups were determined by fitting the model to the conflict data.

### Data sources

This study relied on the analysis of publicly available data. Data on fatalities in the Gaza Strip by age and sex during the ongoing 2023 conflict were obtained by analyzing the list of confirmed deaths reported by the Palestine Ministry of Health for the period October 7, 2023, to October 26, 2023 (17). This database encompassed 6,745 documented deaths after the removal of entries with duplicate national identity numbers (Table 2). This dataset is the most recent detailed information on fatalities available to the authors for this ongoing conflict.

**Table 2:** Distribution of conflict-related deaths by sex and age across five rounds of the Israel-Gaza conflict.

Conflict	2008–2009		2012		2014		2021		2023	
Age group	Males	Females	Males	Females	Males	Females	Males	Females	Males	Females
	*N* = 1,181	*N* = 210	*N* = 146	*N* = 21	*N* = 1,694	*N* = 491	*N* = 175	*N* = 58	*N* = 3,843	*N* = 2,902
	% (95% CI)	% (95% CI)	% (95% CI)	% (95% CI)	% (95% CI)	% (95% CI)	% (95% CI)	% (95% CI)	% (95% CI)	% (95% CI)
0–4	2.1 (1.4–3.0)	1.6 (1.0–2.4)	5.4 (2.5–10.0)	1.8 (0.4–5.2)	3.2 (2.5–4.1)	3.8 (3.0–4.6)	4.3 (2.1–7.8)	1.3 (0.3–3.7)	6.1 (5.5–6.7)	5.4 (4.9–6.0)
5–9	2.2 (1.5–3.1)	1.7 (1.1–2.5)	2.4 (0.7–6.0)	1.2 (0.1–4.3)	4.0 (3.2–4.9)	2.0 (1.4–2.6)	3.9 (1.8–7.2)	2.6 (1.0–5.5)	6.0 (5.4–6.6)	5.6 (5.0–6.1)
10–14	5.7 (4.5–7.0)	2.5 (1.8–3.5)	3.0 (1.0–6.8)	1.2 (0.1–4.3)	4.0 (3.2–4.9)	2.2 (1.7–3.0)	3.9 (1.8–7.2)	3.4 (1.5–6.7)	5.9 (5.4–6.5)	4.8 (4.3–5.4)
15–19	14.2 (12.4–16.1)	1.9 (1.2–2.7)	10.2 (6.0–15.8)	1.2 (0.1–4.3)	10.0 (8.8–11.4)	1.7 (1.2–2.3)	5.6 (3.0–9.4)	2.6 (1.0–5.5)	5.3 (4.8–5.9)	3.8 (3.4–4.3)
20–24	23.3 (21.1–25.6)	1.3 (0.8–2.0)	18.6 (13.0–25.3)	3.0 (1.0–6.8)	19.6 (18.0–21.4)	2.2 (1.6–2.9)	12.9 (8.9–17.9)	1.7 (0.5–4.3)	5.6 (5.0–6.1)	3.5 (3.1–4.0)
25–29	13.2 (11.4–15.0)	0.9 (0.5–1.6)	19.2 (13.5–26.0)	0.6 (0–3.3)	12.9 (11.5–14.4)	2.1 (1.5–2.7)	13.7 (9.6–18.8)	2.6 (1.0–5.5)	5.7 (5.2–6.3)	3.9 (3.4–4.4)
30–34	7.2 (5.9–8.7)	0.8 (0.4–1.4)	10.2 (6.0–15.8)	0.0 (0.0–0.0)	7.0 (6.0–8.2)	1.3 (0.9–1.9)	8.6 (5.3–12.9)	1.7 (0.5–4.3)	6.3 (5.8–7.0)	3.9 (3.5–4.4)
35–39	4.1 (3.1–5.3)	0.5 (0.2–1.0)	4.2 (1.7–8.4)	0.0 (0.0–0.0)	4.5 (3.7–5.5)	1.2 (0.8–1.8)	7.7 (4.6–11.9)	1.7 (0.5–4.3)	4.2 (3.7–4.7)	2.5 (2.2–2.9)
40–44	3.7 (2.8–4.9)	0.9 (0.5–1.6)	3.6 (1.3–7.7)	0.6 (0.0–3.3)	2.7 (2.0–3.4)	1.2 (0.8–1.8)	4.3 (2.1–7.8)	2.1 (0.7–4.9)	2.8 (2.4–3.2)	2.0 (1.6–2.3)
45–49	3.2 (2.4–4.3)	0.7 (0.3–1.3)	3.6 (1.3–7.7)	0.6 (0.0–3.3)	2.4 (1.8–3.1)	1.1 (0.7–1.6)	2.6 (1.0–5.5)	2.1 (0.7–4.9)	2.0 (1.6–2.3)	1.6 (1.3–2.0)
50–54	2.5 (1.8–3.5)	0.3 (0.1–0.7)	3.0 (1.0–6.8)	0.6 (0.0–3.3)	2.5 (1.9–3.3)	1.0 (0.6–1.5)	1.3 (0.3–3.7)	0.0 (0.0–0.0)	1.8 (1.5–2.2)	1.6 (1.3–1.9)
55–59	1.2 (0.7–1.9)	0.5 (0.2–1.0)	1.8 (0.4–5.2)	0.0 (0.0–0.0)	1.6 (1.2–2.3)	0.5 (0.3–0.9)	2.1 (0.7–4.9)	0.9 (0.1–3.1)	1.6 (1.3–2.0)	1.3 (1.0–1.6)
60–64	0.9 (0.5–1.6)	0.5 (0.2–1.0)	1.2 (0.1–4.3)	0.0 (0.0–0.0)	1.1 (0.7–1.6)	0.5 (0.3–1.0)	1.7 (0.5–4.3)	0.0 (0.0–0.0)	1.3 (1.1–1.6)	1.3 (1.0–1.6)
65–69	0.4 (0.2–0.9)	0.2 (0.0–0.6)	0.0 (0.0–0.0)	0.6 (0.0–3.3)	0.7 (0.4–1.2)	0.6 (0.4–1.1)	1.7 (0.5–4.3)	0.9 (0.1–3.1)	1.0 (0.7–1.2)	0.9 (0.7–1.1)
70–74	0.2 (0.0–0.6)	0.3 (0.1–0.7)	0.0 (0.0–0.0)	0.6 (0.0–3.3)	0.4 (0.2–0.7)	0.4 (0.2–0.8)	0.0 (0.0–0.0)	0.4 (0.0–2.4)	0.7 (0.5–1.0)	0.4 (0.3–0.6)
75–79	0.4 (0.1–0.8)	0.2 (0.0–0.6)	1.2 (0.1–4.3)	0.6 (0.0–3.3)	0.6 (0.3–1.0)	0.4 (0.2–0.7)	0.4 (0.0–2.4)	0.0 (0.0–0.0)	0.4 (0.3–0.6)	0.3 (0.2–0.4)
80+	0.5 (0.2–1.0)	0.3 (0.1–0.7)	0.0 (0.0–0.0)	0.0 (0.0–0.0)	0.1 (0.0–0.4)	0.3 (0.1–0.6)	0.4 (0.0–2.4)	0.9 (0.1–3.1)	0.3 (0.2–0.5)	0.4 (0.2–0.5)

CI denotes confidence interval.

This table presents the distribution of conflict-related deaths by age group and sex out of all deaths in each conflict. The distribution of conflict-related deaths by age group, overall, and separately for each of males and females, are provided in Supplementary Tables S1, S2.

Data on fatalities in earlier conflicts were retrieved from the B’Tselem database (18). B’Tselem is an Israeli non-profit organization concerned with documenting human rights violations in the Israeli-occupied Palestinian territories. This comprehensive database covers all conflict-related fatalities that occurred in the Gaza Strip between September 30, 2000, and September 19, 2023, and includes details on whether the fatalities were due to individuals’ potential engagement in combat activities.

The age and sex distributions of fatalities were extracted for specific time periods that defined the armed Israel-Gaza conflicts (Table 2). Specifically, the 2008–2009 conflict spanned from December 27, 2008, to January 18, 2009; the 2012 conflict from November 14, 2012, to November 22, 2012; the 2014 conflict from July 8, 2014, to August 26, 2014; and the 2021 conflict from May 10, 2021, to May 21, 2021.

The aggregate numbers of conflict-related deaths calculated for these periods closely aligned with those reported by the United Nations Office for the Coordination of Humanitarian Affairs (UN OCHA) for these conflicts (19). Here, engagement in combat activities was quantified as a proportion, calculated as the ratio of individuals confirmed to have participated in combat activities or those targeted in killings, to those with available data for this variable.

The demographic distributions of the population of Gaza by age and sex for the years 2009–2021 were obtained from the Palestinian Central Bureau of Statistics (20). This distribution was not available for the year 2023, and was therefore derived by applying the population distribution by age and sex for the year 2022 to the estimated population size of Gaza for 2023 (20). Statistical analyses were performed using Stata/SE version 18.0 (Stata Corporation, College Station, TX, United States).

### Model fitting

The hazard rates δ and μ were derived by fitting the sex and age distributions of conflict-related deaths in each round of conflict, along with the aggregate numbers of conflict-related deaths. Model fitting employed a nonlinear least-square fitting method, minimizing the sum of squares between all data points and model predictions. This technique, along with all modeling analyses, was implemented in MATLAB (21) using the Nelder–Mead simplex algorithm (22).

### Uncertainty analysis

An uncertainty analysis was conducted to estimate the range of uncertainty in the projected outcomes. This process involved randomly sampling data points from the 95% confidence intervals of the sex and age distributions of conflict-related deaths. The model was then refitted to these data points. This process was repeated 1,000 times to generate distributions for the estimated outcomes. Subsequently, these distributions were utilized to derive the means and 95% uncertainty intervals (UIs).

### Sensitivity analysis

The demographic composition of the Gaza population is skewed towards a younger age, with over 50% of Gazans being under 20 years old (20). Consequently, the model’s estimates pertaining to older age groups are less certain due to the sparse numbers of fatalities in these categories. Moreover, the assumption that the combatant population comprises males between 15 and 64 years old might not accurately reflect reality, as males between 50 and 64 are likely infrequently involved in combat. Consequently, a sensitivity analysis was conducted, wherein all model estimates were recalculated, assuming the combatant population consists only of males between 15 and 49 years old.

## Results

**Figure 1:**
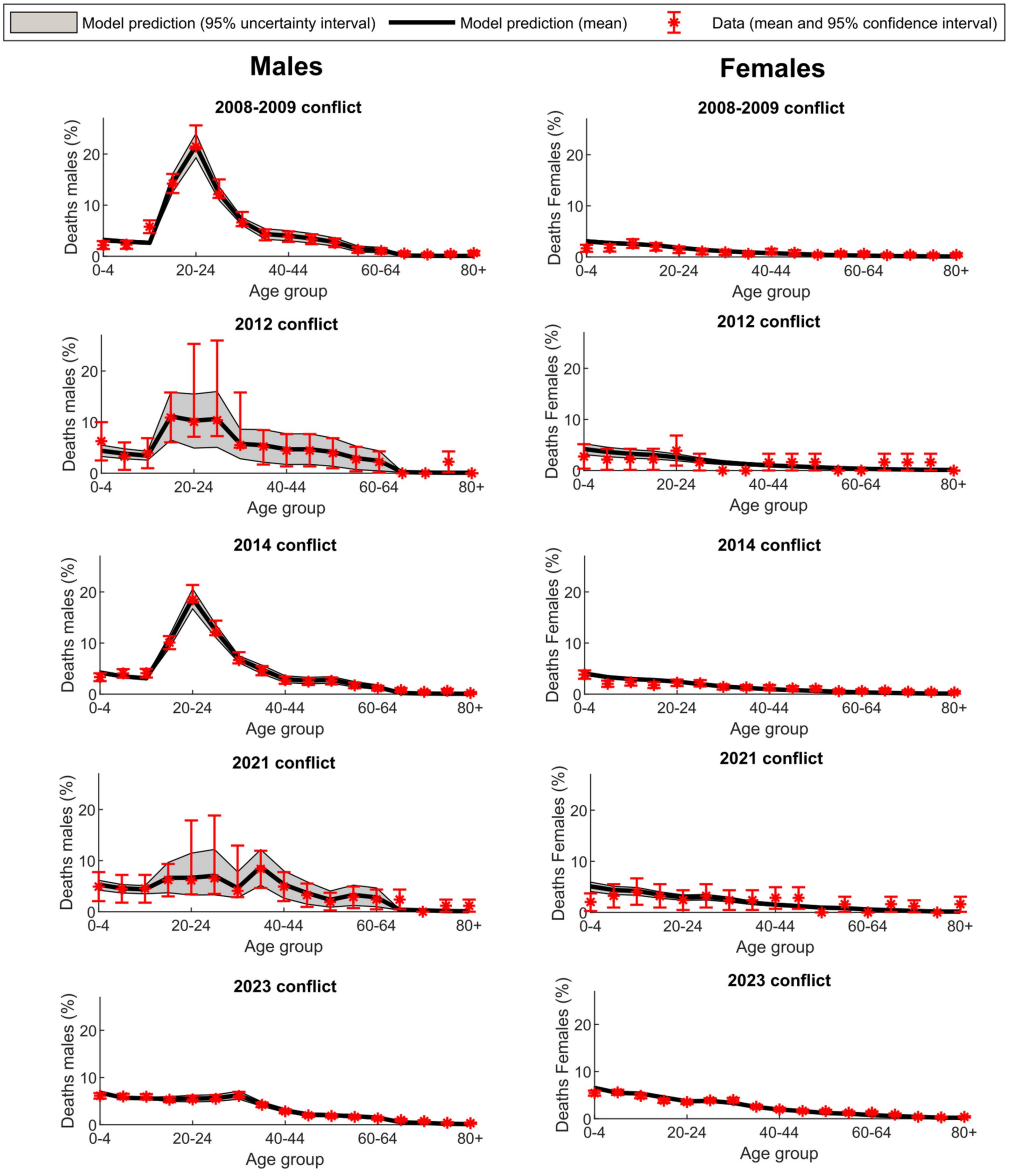
Model fit to conflict-related mortality data for males and females, including both combatant and civilian deaths, across age groups in the five analyzed Israel-Gaza conflicts. Proportions are presented as a percentage of total deaths in each conflict.

Figure 1 illustrates the model’s fit to conflict-related death data, encompassing combatant and civilian deaths, for both males and females across the different age groups in the five examined conflicts. The figure displays the proportions of deaths (not the absolute number of deaths) by age and sex in each of the conflicts. The model exhibited a good fit to the data for the 2008–2009, 2014, and 2023 conflicts. However, statistical uncertainties were high in the 2012 and 2021 conflicts, primarily due to the relatively small number of fatalities in these two conflicts (refer to Table 2). This underscores that the statistical methodology introduced here is most effective for examining large-scale conflicts, as it provides statistical population-level evidence rather than individual-level evidence.

Notably, the proportion of female deaths across age groups was similar across all conflicts and closely mirrored the age structure of the population, aligning with the assumption of a μ that is largely independent of age, as assumed in this modeling approach, and that females are not engaged in combat activities. Meanwhile, the distribution of male deaths exhibited a peak among young adults, consistent with young adult men being the primary combatant age group in the population.

**Figure 2:**
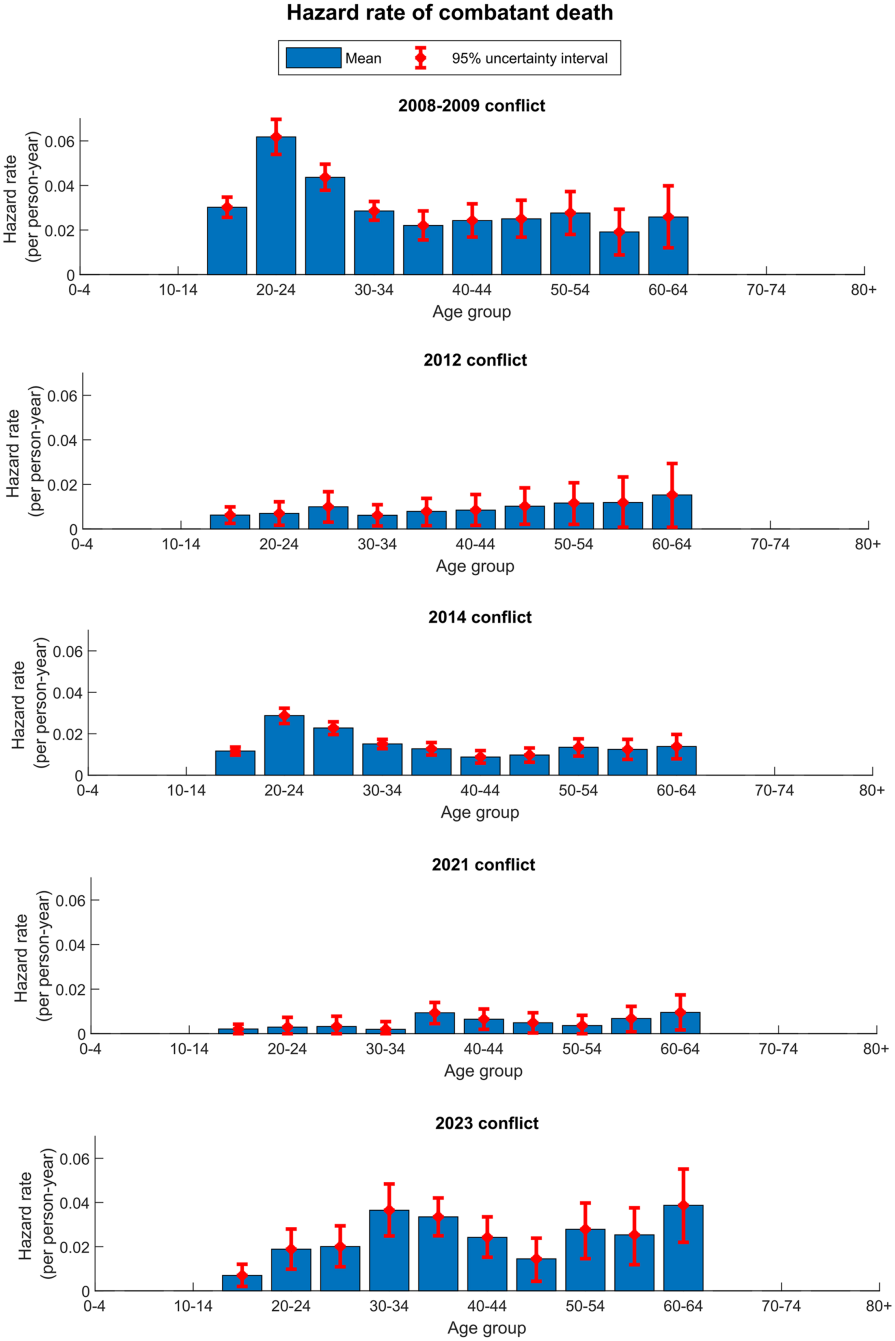
The model-estimated δ, representing the hazard rate of combatant death (force of combatant killing), plotted against age for the male population in the five Israel-Gaza conflicts. Females were assumed not engaged in combat.

Figure 2 shows the model-estimated δ, representing the force of combatant killing, plotted against age for the male population. The variations in δ across conflicts demonstrate the variation in the intensity of combat activities. In the 2012 and 2021 conflicts, δ was relatively small with wide uncertainty intervals, attributed to the relatively small scale of these two conflicts. In contrast, δ in the 2008–2009, 2014, and 2023 conflicts was relatively large, reflecting the intensities and scales of these conflicts. Moreover, in the 2008–2009 and 2014 conflicts, unlike in the 2023 conflict, a noticeable peak among young adult men in their 20s is evident. This observation aligns with individuals in their 20s being the predominant age group within Gaza’s combatant force.

**Figure 3:**
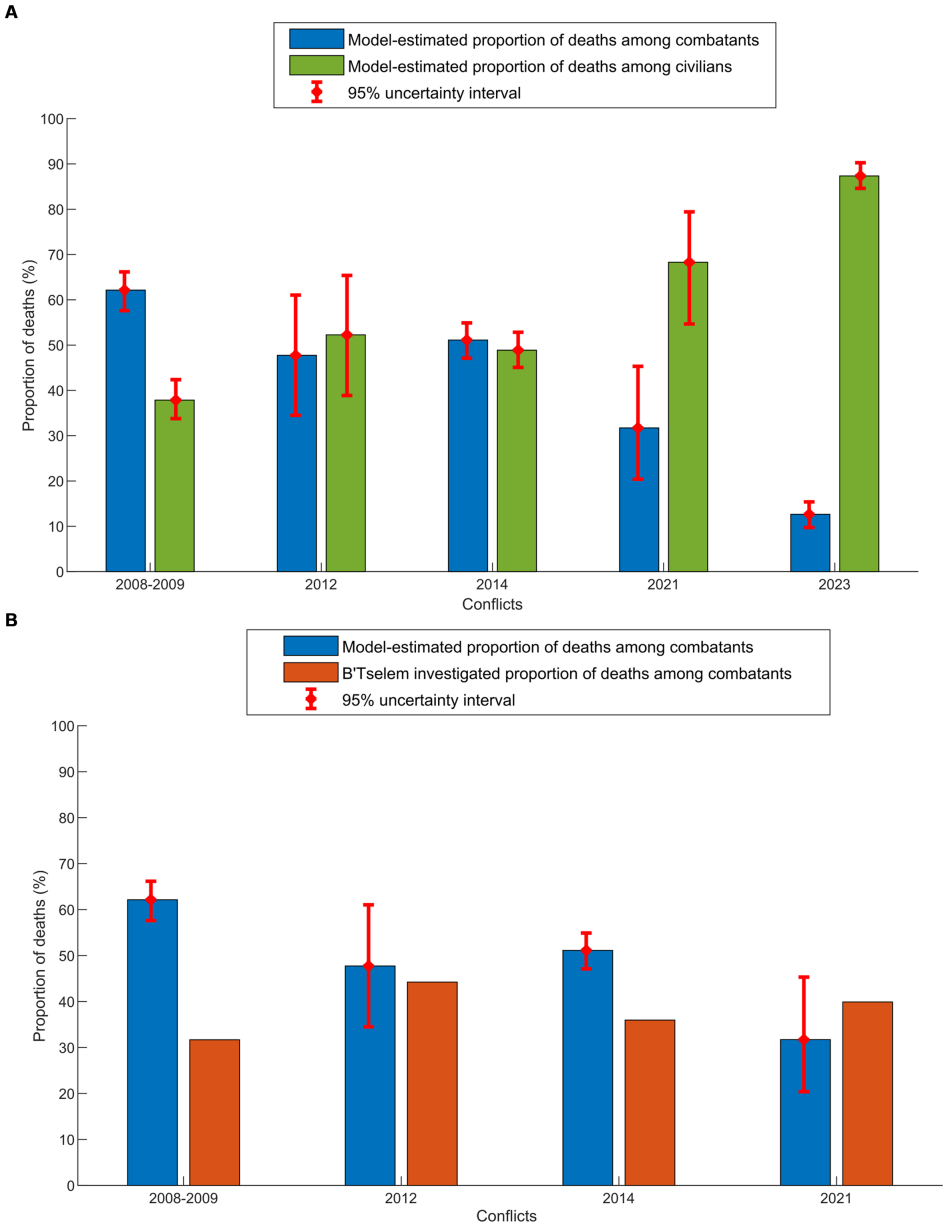
**(A)** Model-estimated proportions of combatant and civilian deaths in the five Israel-Gaza conflicts. **(B)** Comparison between the model-estimated proportion of combatant deaths and the actual proportion estimated based on investigating each death by B’Tselem (18). No B’Tselem investigation is available for the 2023 conflict.

Figure 3A presents the model-estimated proportion of deaths categorized as combatants versus civilians. In the 2008–2009 conflict, the proportion of deaths attributed to combatants was close to twice that of civilians. In the 2012 and 2014 conflicts, the proportion of deaths categorized as combatants versus civilians was comparable, with a slight elevation for civilians in 2012 and a slight increase for combatants in 2014. In the 2021 conflict, the proportion of deaths attributed to combatants was approximately half that of civilians. The 2023 conflict stands out as singular and distinct from all previous conflicts, with estimated deaths among civilians several times higher than that among combatants.

Figure 3B illustrates a comparison between the model-estimated proportion of deaths categorized as combatants and the actual estimation based on investigating each death by B’Tselem (18). In the conflicts with narrow uncertainty intervals in estimates, the 2008–2009 and 2014 conflicts, the model-estimated proportion of combatant deaths substantially exceeded B’Tselem’s estimated proportion (18). The reason for this overestimation is likely related to the implicit assumption in the model that the increase in the male hazard rate of death relative to females is solely attributed to participation in combat. However, young adult men may have a higher civilian hazard rate of death than females because they are more likely to be the primary caretakers for their families, making them more susceptible to conflict-related fatalities while engaged in civilian activities like procuring essential needs. This overestimation of combatants in the model suggests that evidence generated by the present method is likely to be conservative and may underestimate the toll of conflicts on civilians.

**Figure 4:**
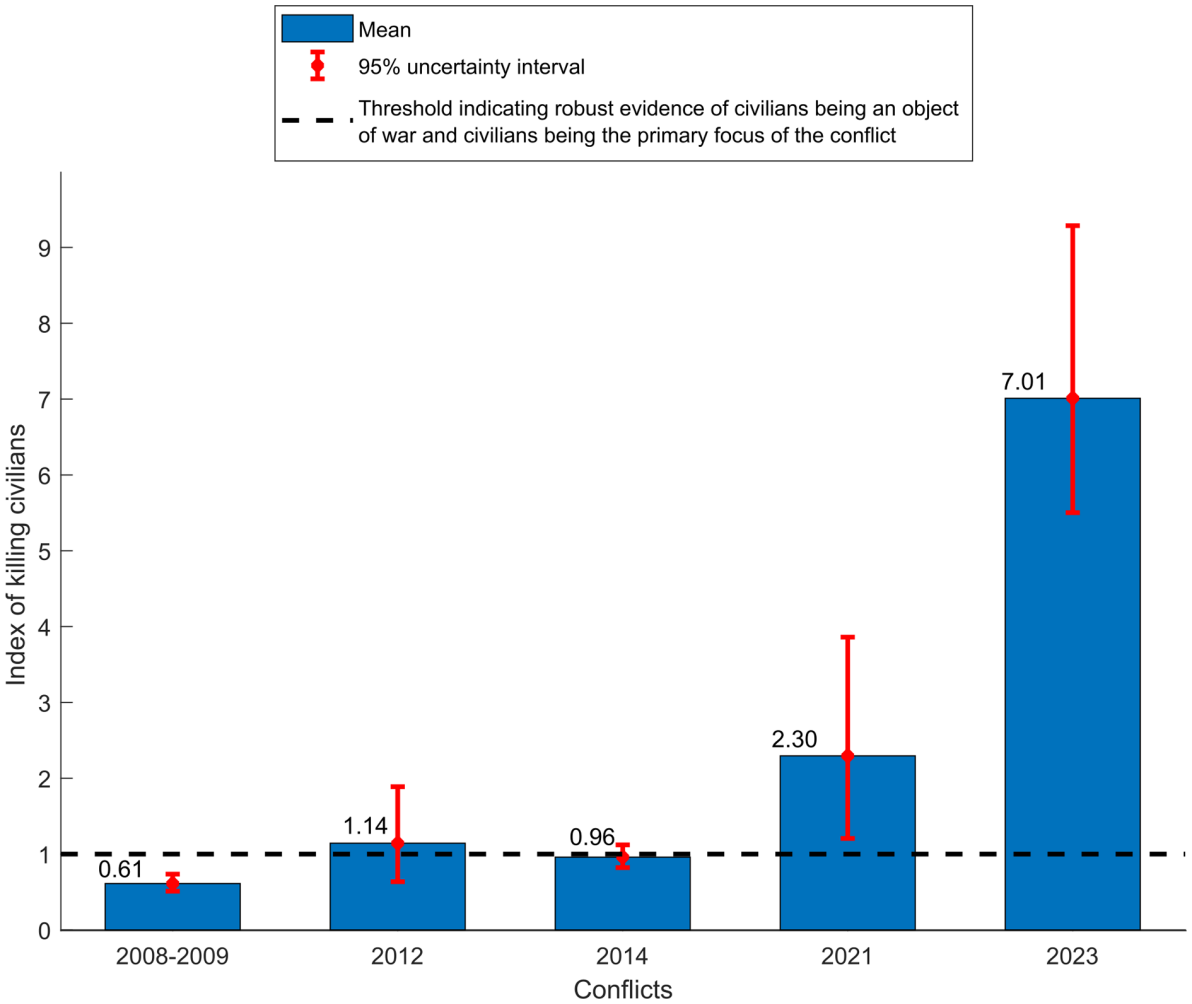
Index of killing civilians (n) in the five Israel-Gaza conflicts.

Figure 4 displays n, the index of killing civilians, for the examined conflicts. In the 2008–2009 and 2014 conflicts, n was 0.61 (95% UI: 0.51–0.74) and 0.96 (95% UI: 0.82–1.12), respectively. These values indicate strong evidence for civilians being an object of war (Table 1), but that the focus of these conflicts was still more on combatants than civilians. In the 2012 and 2021 conflicts, the uncertainty intervals were wide in the n estimates, but the results were still consistent with strong, if not robust, evidence for civilians being an object of war. In the 2023 conflict, n was 7.01 (95% UI: 5.50–9.29), much larger than 1, signifying robust evidence for civilians being an object of war, and that civilians are the primary focus of the conflict rather than combatants.

**Figure 5:**
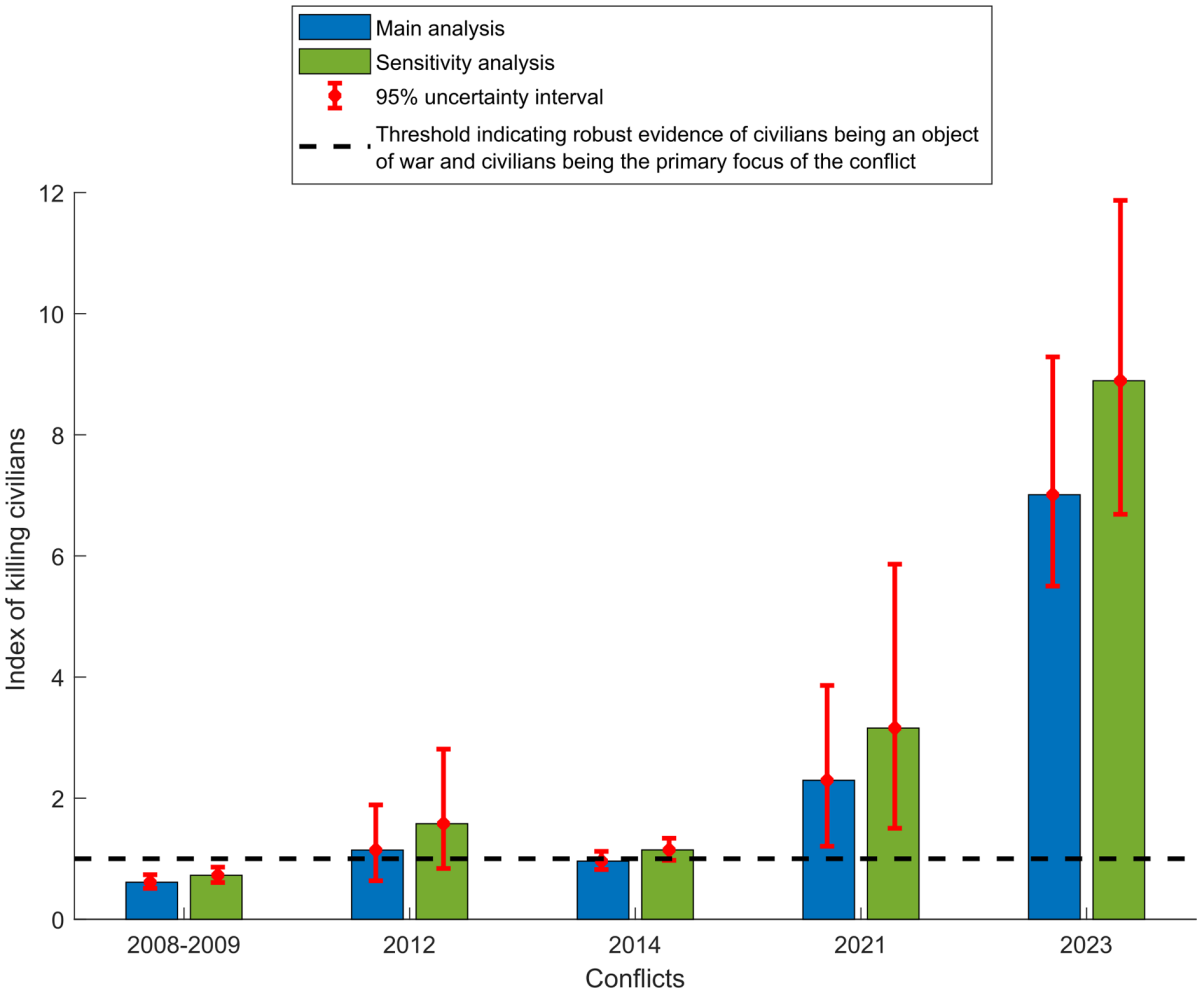
Comparison of the results for n, the index of killing civilians, in the sensitivity analysis assuming the combatant population consists only of males between 15 and 49 years old, with the results of the main analysis, assuming the combatant population consists of males between 15 and 64 years old, across examined conflicts.

Figure 5 presents a comparison of the results for n in the sensitivity analysis, assuming the combatant population consists only of males between 15 and 49 years old, with the results of the main analysis across examined conflicts. Both the sensitivity and main analysis results confirmed similar findings, indicating that model uncertainties arising from the smaller demographics of the older population are not likely to impact the study’s conclusions. The analysis also indicated that incorporating individuals aged 50–64 into the combatant population led to more conservative estimates, thereby underestimating the impact of the conflict on civilians.

## Discussion

We introduced an approach for the quantitative investigation of civilian deaths in conflicts. This methodology represents a step toward developing a broader class of methods tailored for scrutinizing civilian casualties in conflict situations, complementing other approaches, such as the investigation of conflict incidents, to provide evidence of whether civilians were a target of war activity. Further refinement will enhance the approach and broaden its applicability. This includes refining the specific criteria for categorizing conflicts using the index of killing civilians (Table 1), by setting it based on in-depth investigations of historical conflicts.

Refinement is also needed to address potential variations in the civilian conflict-related hazard rate of death based on sex and age. This may involve incorporating additional data sources, not available in this study, into the model fitting process. In a broader context, there is a need to establish international databases for conflicts, with data stratified by relevant factors such as age and sex, to facilitate the application of analytical methods like the one introduced here. Existing databases, such as the Uppsala Conflict Data Program (23) and the Global Burden of Disease study (24), could also be expanded to include such detailed information for each conflict or specific round of conflict.

Applying the introduced approach to Gaza deaths in five distinct rounds of the Israel-Gaza conflict revealed key findings. Firstly, the 2023 conflict stands out as distinctly different from all preceding rounds of conflict. The results indicate that civilians were and still are an object of war, and importantly, the primary focus of this specific conflict—a departure from the pattern observed in earlier rounds of the conflict. The findings do not align with civilian casualties being attributed to collateral damage, prompting questions about the intent behind the combat activities that resulted in such extensive civilian mortality.

Secondly, the findings suggest a progressive shift in Israel’s undisclosed rules of engagement over time, transitioning towards higher acceptance of casualties among civilians. The index of killing civilians demonstrated an increasing trend across the conflict rounds (Figure 4), with a major surge in the 2023 conflict, reflecting a pronounced change in rules of engagement. The sharp increase reflected a massive rise in the civilian hazard rate of death relative to the combatant hazard rate of death. Reports from human rights organizations and news sources substantiate these findings, confirming unprecedented documented attacks on civilians (25, 26). These attacks have resulted in mass civilian casualties, the obliteration of entire families, and extensive destruction of residential neighborhoods (25).

This study has limitations. While we introduced, to our knowledge, a novel approach to investigating civilian deaths in conflicts, further refinement of the model can enhance the methodology’s applicability and improve its capacity to generate precise estimations. Nonetheless, the results from applying the model to the Israel-Gaza conflicts are clear-cut, particularly in highlighting stark differences between the 2023 conflict and earlier conflicts. The uncertainty and sensitivity analyses further supported the robustness of the findings. This suggests that any future refinement is unlikely to alter these findings.

The study relies on various data sources, including publicly available information and data from independent organizations. Certain datasets, particularly those related to death counts, were collected during periods of conflict. While these sources are widely utilized and acknowledged by international organizations, they have not been formally published in scientific literature. Given that independent validation of such data may not always be feasible, the data may be susceptible to distortion in a war context.

While the data inputs for the conflict rounds preceding 2023 are complete and well-documented, the ongoing nature of the 2023 conflict means that its data are neither final nor complete, and are subject to potential changes as the conflict unfolds, particularly in relation to capturing counts of deaths and the age and sex distributions of fatalities.

In conflict zones, accurately obtaining data on civilian versus combatant casualties presents challenges due to the chaotic nature of conflicts and a lack of systematic data collection (12). Parties involved may intentionally distort casualty figures, complicating the determination of the true scale of losses. The infrastructure for accurate reporting and verification often becomes compromised, further impeding data collection efforts. Consequently, casualty estimates typically depend on incomplete information from various sources, introducing potential limitations in data usage. Nonetheless, this underscores the need for innovative methods to estimate civilian versus combatant casualties, as proposed in this study.

The model’s estimates for older age groups exhibit wide uncertainty intervals, primarily attributable to the sparse numbers of fatalities within these small age demographics. Minor variations in the number of fatalities within the older age groups can significantly impact the model’s estimates for these specific cohorts. Nevertheless, the model’s predictions, being based on the total population, are not likely to be influenced by these uncertainties. This assertion is further supported by the results of the conducted sensitivity analysis (Figure 5). Despite these limitations, the model yielded informative results in estimating effects, and there are reasons to consider the model estimates as conservative, potentially underestimating the true toll of the conflicts on civilians.

In conclusion, this study introduced and implemented a quantitative approach to investigate civilian deaths in conflicts, with a specific application examining Gaza deaths in five Israel-Gaza conflicts. The approach carries the potential for establishing statistical evidence, complementing traditional forms of evidence, in investigating civilian deaths in conflicts. The findings highlighted the distinctive nature of the 2023 Israel-Gaza conflict, characterized by extensive toll on civilians, with civilians being even identified as the primary focus of the conflict. The results also suggested a progressive shift in Israel’s rules of engagement over time, moving towards higher acceptance of casualties among civilians.

## Data availability statement

The original contributions presented in the study are included in the article/Supplementary material, further inquiries can be directed to the corresponding author.

## Ethics statement

This study relied on the analysis of publicly available data, exempt from regulatory review and approvals.

## Author contributions

HA: Writing – review & editing. HC: Writing – review & editing. LA-R: Writing – original draft, Writing – review & editing.
