# Supplementary material for: Comparative analysis and evolution of civilian versus combatant mortality ratios in Israel-Gaza conflicts, 2008–2023
Source: Front Public Health. 2024 Jun 25;12:1359189. doi: 10.3389/fpubh.2024.1359189 (PMC11231088; doi:10.3389/fpubh.2024.1359189)
Supplement: Supplementary file 1 [file Table_1.DOCX]

**Supplementary Appendix**

**Table S1.** Distribution of conflict-related deaths by age, overall, and separately for each of males and females, in the 2008-2009, 2012, and 2014 rounds of the Israel-Gaza conflict.

| **Conflict** | **2008-2009** | | | **2012** | | | **2014** | | |
| --- | --- | --- | --- | --- | --- | --- | --- | --- | --- |
| **Age group** | **Males** | **Females** | **Overall** | **Males** | **Females** | **Overall** | **Males** | **Females** | **Overall** |
|  | N=1,181 | N=210 | N=1,388 | N=146 | N=21 | N=167 | N=1,694 | N=491 | N=2,185 |
|  | % (95% CI) | % (95% CI) | % (95% CI) | % (95% CI) | % (95% CI) | % (95% CI) | % (95% CI) | % (95% CI) | % (95% CI) |
| **0-4** | 2.5 (1.7-3.5) | 10.5 (6.7-15.4) | 3.7 (2.7-4.8) | 6.2 (2.9-11.4) | 14.3 (3.0-36.3) | 7.2 (3.8-12.2) | 4.2 (3.3-5.3) | 16.7 (13.5-20.3) | 7.0 (6.0-8.2) |
| **5-9** | 2.5 (1.7-3.6) | 11.0 (7.1-16.0) | 3.8 (2.9-5.0) | 2.7 (0.8-6.9) | 9.5 (1.2-30.4) | 3.6 (1.3-7.7) | 5.1 (4.1-6.3) | 8.8 (6.4-11.6) | 5.9 (5.0-7.0) |
| **10-14** | 6.7 (5.3-8.3) | 16.7 (11.9-22.4) | 8.2 (6.8-9.8) | 3.4 (1.1-7.8) | 9.5 (1.2-30.4) | 4.2 (1.7-8.4) | 5.2 (4.2-6.4) | 10.0 (7.5-13.0) | 6.3 (5.3-7.4) |
| **15-19** | 16.7 (14.6-18.9) | 12.4 (8.2-17.6) | 16.1 (14.2-18.1) | 11.6 (6.9-18.0) | 9.5 (1.2-30.4) | 11.4 (7.0-17.2) | 12.9 (11.4-14.6) | 7.5 (5.4-10.2) | 11.7 (10.4-13.1) |
| **20-24** | 27.4 (24.9-30.1) | 8.6 (5.2-13.2) | 24.6 (22.4-27.0) | 21.2 (14.9-28.8) | 23.8 (8.2-47.2) | 21.6 (15.6-28.6) | 25.3 (23.3-27.5) | 9.6 (7.1-12.5) | 21.8 (20.1-23.6) |
| **25-29** | 15.5 (13.5-17.7) | 6.2 (3.3-10.4) | 14.1(12.3-16.1) | 21.9 (15.5-29.5) | 4.8 (0.1-23.8) | 19.8 (14.0-26.6) | 16.6 (14.9-18.5) | 9.2 (6.8-12.1) | 15.0 (13.5-16.5) |
| **30-34** | 8.5 (6.9-10.2) | 5.2 (2.6-9.2) | 8.0 (6.6-9.6) | 11.6 (6.9-18.0) | 0.0 (0.0-16.1) | 10.2 (6.0-15.8) | 9.1 (7.8-10.6) | 5.9 (4.0-8.4) | 8.4 (7.2-9.6) |
| **35-39** | 4.8 (3.7-6.2) | 3.3 (1.4-6.7) | 4.6 (3.6-5.9) | 4.8 (1.9-9.6) | 0.0 (0.0-16.1) | 4.2 (1.7-8.4) | 5.8 (4.8-7.1) | 5.5 (3.7-7.9) | 5.8 (4.8-6.8) |
| **40-44** | 4.4 (3.3-5.7) | 6.2 (3.3-10.4) | 4.7 (3.6-5.9) | 4.1 (1.5-8.7) | 4.8 (0.1-23.8) | 4.2 (1.7-8.4) | 3.4 (2.6-4.4) | 5.5 (3.7-7.9) | 3.9 (3.1-4.8) |
| **45-49** | 3.8 (2.8-5.1) | 4.8 (2.3-8.6) | 4.0 (3.0-5.1) | 4.1 (1.5-8.7) | 4.8 (0.1-23.8) | 4.2 (1.7-8.4) | 3.1 (2.3-4.0) | 4.7 (3.0-6.9) | 3.4 (2.7-4.3) |
| **50-54** | 3.0 (2.0-4.1) | 1.9 (0.5-4.8) | 2.8 (2.0-3.8) | 3.4 (1.1-7.8) | 4.8 (0.1-23.8) | 3.6 (1.3-7.7) | 3.2 (2.5-4.2) | 4.5 (2.8-6.7) | 3.5 (2.8-4.4) |
| **55-59** | 1.4 (0.8-2.2) | 3.3 (1.4-6.7) | 1.7 (1.1-2.5) | 2.1 (0.4-5.9) | 0.0 (0.0-16.1) | 1.8 (0.4-5.2) | 2.1 (1.5-2.9) | 2.2 (1.1-4.0) | 2.2 (1.6-2.8) |
| **60-64** | 1.1 (0.6-1.9) | 3.3 (1.4-6.7) | 1.4 (0.9-2.2) | 1.4 (0.2-4.9) | 0.0 (0.0-16.1) | 1.2 (0.1-4.3) | 1.4 (0.9-2.1) | 2.4 (1.3-4.2) | 1.6 (1.2-2.3) |
| **65-69** | 0.5 (0.2-1.1) | 1.4 (0.3-4.1) | 0.6 (0.3-1.2) | 0.0 (0.0-2.5) | 4.8 (0.1-23.8) | 0.6 (0.0-3.3) | 0.9 (0.5-1.5) | 2.9 (1.6-4.7) | 1.4 (0.9-1.9) |
| **70-74** | 0.3 (0.1-0.7) | 1.9 (0.5-4.8) | 0.5 (0.2-1.0) | 0.0 (0.0-2.5) | 4.8 (0.1-23.8) | 0.6 (0.0-3.3) | 0.5 (0.2-0.9) | 1.8 (0.8-3.4) | 0.8 (0.5-1.2) |
| **75-79** | 0.4 (0.1-1.0) | 1.4 (0.3-4.1) | 0.6 (0.2-1.1) | 1.4 (0.2-4.9) | 4.8 (0.1-23.8) | 1.8 (0.4-5.2) | 0.8 (0.4-1.3) | 1.6 (0.7-3.2) | 1.0 (0.6-1.5) |
| **80+** | 0.6 (0.2-1.2) | 1.9 (0.5-4.8) | 0.6 (0.2-1.1) | 0.0 (0.0-2.5) | 0.0 (0.0-16.1) | 0.0 (0.0-2.2) | 0.2 (0.0-0.5) | 1.2 (0.4-2.6) | 0.4 (0.2-0.8) |

CI denotes confidence interval.

**Table S2.** Distribution of conflict-related deaths by age, overall, and separately for each of males and females, in the 2021 and 2023 rounds of the Israel-Gaza conflict.

| **Conflict** | **2021** | | | **2023** | | |
| --- | --- | --- | --- | --- | --- | --- |
| **Age group** | **Males** | **Females** | **Overall** | **Males** | **Females** | **Overall** |
|  | N=175 | N=58 | N=233 | N=3,843 | N-2,902 | N=6,745 |
|  | % (95% CI) | % (95% CI) | % (95% CI) | % (95% CI) | % (95% CI) | % (95% CI) |
| **0-4** | 5.7 (2.8-10.3) | 5.2 (1.1-14.4) | 5.6 (3.0-9.4) | 10.7 (9.7-11.7) | 12.5 (11.4-13.8) | 11.5 (10.7-12.3) |
| **5-9** | 5.1 (2.4-9.5) | 10.3 (3.9-21.2) | 6.4 (3.6-10.4) | 10.5 (9.5-11.5) | 13.0 (11.8-14.2) | 11.5 (10.8-12.3) |
| **10-14** | 5.1 (2.4-9.5) | 13.8 (6.1-25.4) | 7.3 (4.3-11.4) | 10.4 (9.4-11.4) | 11.2 (10.1-12.4) | 10.7 (10.0-11.5) |
| **15-19** | 7.4 (4.0-12.4) | 10.3 (3.9-21.2) | 8.2 (5.0-12.4) | 9.3 (8.4-10.3) | 8.8 (7.8-9.9) | 9.1 (8.4-9.8) |
| **20-24** | 17.1 (11.9-23.6) | 6.9 (1.9-16.7) | 14.6 (10.3-19.8) | 9.8 (8.9-10.8) | 8.2 (7.2-9.3) | 9.1 (8.4-9.8) |
| **25-29** | 18.3 (12.9-24.8) | 10.3 (3.9-21.2) | 16.3 (11.8-21.7) | 10.0 (9.1-11.0) | 9.0 (8.0-10.1) | 9.6 (8.9-10.3) |
| **30-34** | 11.4 (7.1-17.1) | 6.9 (1.9-16.7) | 10.3 (6.7-14.9) | 11.1 (10.2-12.2) | 9.1 (8.1-10.2) | 10.3 (9.6-11.0) |
| **35-39** | 10.3 (6.2-15.8) | 6.9 (1.9-16.7) | 9.4 (6.0-13.9) | 7.4 (6.6-8.2) | 5.9 (5.1-6.8) | 6.7 (6.1-7.4) |
| **40-44** | 5.7 (2.8-10.3) | 8.6 (2.9-19.0) | 6.4 (3.6-10.4) | 4.9 (4.3-5.7) | 4.5 (3.8-5.4) | 4.8 (4.3-5.3) |
| **45-49** | 3.4 (1.3-7.3) | 8.6 (2.9-19.0) | 4.7 (2.4-8.3) | 3.4 (2.9-4.1) | 3.8 (3.1-4.6) | 3.6 (3.2-4.1) |
| **50-54** | 1.7 (0.4-4.9) | 0.0 (0.0-6.2) | 1.3 (0.3-3.7) | 3.2 (2.7-3.8) | 3.7 (3.0-4.4) | 3.4 (3.0-3.9) |
| **55-59** | 2.9 (0.9-6.5) | 3.4 (0.4-11.9) | 3.0 (1.2-6.1) | 2.8 (2.3-3.4) | 2.9 (2.3-3.6) | 2.9 (2.5-3.3) |
| **60-64** | 2.3 (0.6-5.7) | 0.0 (0.0-6.2) | 1.7 (0.5-4.3) | 2.3 (1.9-2.8) | 3.0 (2.4-3.6) | 2.6 (2.2-3.0) |
| **65-69** | 2.3 (0.6-5.7) | 3.4 (0.4-11.9) | 2.6 (0.9-5.5) | 1.7 (1.3-2.2) | 2.0 (1.5-2.6) | 1.8 (1.5-2.2) |
| **70-74** | 0.0 (0.0-2.1) | 1.7 (0.0-9.2) | 0.4 (0.0-2.4) | 1.3 (0.9-1.7) | 0.9 (0.6-1.3) | 1.1 (0.9-1.4) |
| **75-79** | 0.6 (0.0-3.1) | 0.0 (0.0-6.2) | 0.4 (0.0-2.4) | 0.7 (0.4-1.0) | 0.6 (0.4-1.0) | 0.7 (0.5-0.9) |
| **80+** | 0.6 (0.0-3.1) | 3.4 (0.4-11.9) | 1.3 (0.3-3.7) | 0.5 (0.3-0.8) | 0.9 (0.6-1.3) | 0.7 (0.5-0.9) |

CI denotes confidence interval.
